# The Role of Zinc Against Bacterial Infections in Neonates, Children, and Adults: A Scoping Review from the Available Evidence of Randomized Controlled Trials About Zinc Supplementation to New Research Opportunities

**DOI:** 10.3390/antibiotics15010066

**Published:** 2026-01-08

**Authors:** Domenico Umberto De Rose, Nicola Mirotta, Andrea Dotta, Guglielmo Salvatori, Maria Paola Ronchetti, Laura Campogiani, Francesca Ceccherini-Silberstein, Marco Iannetta

**Affiliations:** 1 Neonatal Intensive Care Unit, “Bambino Gesù” Children’s Hospital IRCCS, 00165 Rome, Italy; andrea.dotta@opbg.net (A.D.); guglielmo.salvatori@opbg.net (G.S.); mariapaola.ronchetti@opbg.net (M.P.R.); 2 Unità Valorizzazione della Ricerca, Consiglio Nazionale delle Ricerche, Piazzale Aldo Moro, 7-, 00185 Rome, Italy; nicola.mirotta@cnr.it; 3 Infectious Disease Clinic, Policlinico “Tor Vergata”, 00133 Rome, Italy; lauracampg@gmail.com (L.C.); marco.iannetta@uniroma2.it (M.I.); 4 Department of Experimental Medicine, “Tor Vergata” University of Rome, 00133 Rome, Italy; ceccherini@med.uniroma2.it; 5 Department of System Medicine, “Tor Vergata” University of Rome, 00133 Rome, Italy

**Keywords:** zinc supplementation, bacterial infections, RCT, antimicrobial resistance, nanoparticles, zinc oxide, host–pathogen interactions, nanotechnology

## Abstract

(1) **Background**: Zinc is an essential micronutrient involved in immune regulation, epithelial barrier integrity, and the host response to bacterial infections. However, the clinical benefits of zinc supplementation across different age groups remain uncertain, with heterogeneous findings and variable dosing strategies reported in the literature. (2) **Objectives**: To map and summarize randomized controlled trials (RCTs) evaluating zinc supplementation (either as treatment or prophylaxis) for bacterial infection outcomes in neonates, children, and adults, and to identify gaps requiring further research, including the use of zinc-based nanoparticles. (3) **Eligibility Criteria**: We included English-language RCTs that evaluated zinc supplementation and reported clinical outcomes related to bacterial infections. Observational studies, trials without infection-related outcomes, and studies not involving human participants were excluded. (4) **Sources of Evidence:** A MEDLINE (PubMed) search was conducted from 2000 to 1 November 2025 using predefined keywords related to zinc supplementation, neonates, children, adults, and bacterial infections. Reference lists of eligible articles were screened to identify additional studies. (5) **Charting Methods:** Data were charted for each included study, including population characteristics, zinc dosing and regimen, type of supplementation (therapeutic or prophylactic), main infection-related outcomes, and key findings. Data charting was performed independently and verified within the research team. (6) **Results**: A total of 51 RCTs were included: 10 in neonates, 32 in children, and 9 in adults. In neonates, therapeutic zinc supplementation as an adjunct to antibiotics showed heterogeneous results, with some studies reporting reductions in morbidity, inflammatory markers or mortality, while others found no significant differences in clinical outcomes. In children, zinc supplementation consistently reduced the duration and severity of diarrheal episodes and, in several trials, improved the resolution of respiratory infections. In adults, the evidence was limited but suggested potential benefits in selected populations, such as burn patients or those with zinc deficiency or immunologic dysfunction. Variability in zinc dosage, treatment duration, and outcome definitions limits direct comparison across studies. (7) **Conclusions:** Zinc supplementation appears to provide benefits in neonates and children, whereas evidence in adults remains mixed and inconclusive. Standardized, well-powered RCTs are needed to define optimal dosing strategies, identify populations most likely to benefit, and clarify the mechanisms underlying zinc’s anti-infective effects. Future research should consider the use of zinc oxide nanoparticles (ZnO-NPs) demonstrated broad-spectrum antimicrobial activity and potential synergy with antibiotics, although clinical data remain still limited.

## 1. Introduction

**Figure 1:**
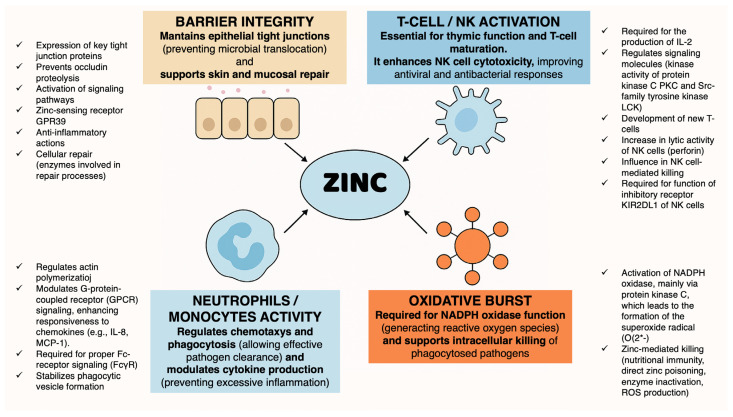
Schematic representation of the key immunological functions supported by zinc.

Zinc is a key element for numerous enzymes and transcription factors, and zinc deficiency is associated with major clinical consequences, including growth retardation, impaired cell-mediated immunity, and cognitive impairment [1]. Zinc deficiency remains a major global health problem, affecting approximately 17% of the world’s population, with the highest burden in low- and middle-income countries. Among children under five years of age, the estimated prevalence ranges from 20% to over 30% depending on geographic region and dietary patterns [2]. Acquired zinc deficiency is characterized by clinical features such as diarrhea, recurrent infections, and skin lesions [3]. Zinc plays a central role in immune function, epithelial integrity, and inflammatory modulation (Figure 1) [4,5,6,7,8]; consequently, zinc deficiency has been associated with increased susceptibility to respiratory infections, diarrhea, and impaired host defense. Current epidemiological data highlight the clinical relevance of understanding zinc status in relation to infection risk and treatment outcomes [9].

Beyond its essential role in host immune defense, zinc is also a critical micronutrient for bacterial physiology, as it is required for the activity of multiple bacterial enzymes and regulatory systems. During infection, the host actively modulates zinc availability as part of nutritional immunity, while pathogens have evolved mechanisms to acquire zinc under restrictive conditions. This dual role at the host–pathogen interface makes zinc supplementation a biologically plausible intervention to modulate infection outcomes, particularly in populations at higher risk of zinc deficiency such as neonates, children, and vulnerable adult groups. During infections, total plasma zinc levels decrease in order to restrict bacterial access to zinc. This process is mainly mediated by the upregulation of the zinc transporter ZIP14 through an interleukin-6–mediated pathway. This response occurs rapidly (within a few hours), resulting in reduced plasma zinc levels and concomitant zinc accumulation in the liver [10,11].

Different bacterial species have developed specific strategies to overcome zinc limitation. For example, Neisseria species (*N. gonorrhoeae* and *N. meningitidis*) express dedicated zinc import systems, including ZnuC, ZnuB, and ZnuA. Znu-like systems were also found in various bacteria, including *E. coli* and *K. pneumoniae* [11].

Randomized controlled trials (RCTs) evaluating zinc supplementation have been conducted in neonates, children, and adults, but differ widely in population characteristics, dosing regimens, clinical settings, and infection-related outcomes. This variability has resulted in fragmented evidence and challenges in drawing generalizable conclusions.

Zinc requirements, baseline deficiency prevalence, immune system maturation, and infection outcomes vary substantially across the life course. Neonates and young children are particularly vulnerable to zinc deficiency and infectious morbidity, whereas adults may exhibit heterogeneous responses depending on nutritional status, comorbidities, and immune dysfunction. For this reason, a unified but age-stratified analysis is required to comprehensively evaluate the effects of zinc supplementation on bacterial infections across different stages of life.

This scoping review aimed to systematically map randomized controlled trials evaluating zinc supplementation for bacterial infection outcomes in neonates, children, and adults. By synthesizing age-stratified evidence, we sought to identify consistent effects, sources of heterogeneity, and knowledge gaps, including emerging research directions related to antimicrobial resistance and zinc-based nanotechnologies.

## 2. Results

### 2.1. Study Description

**Figure 2:**
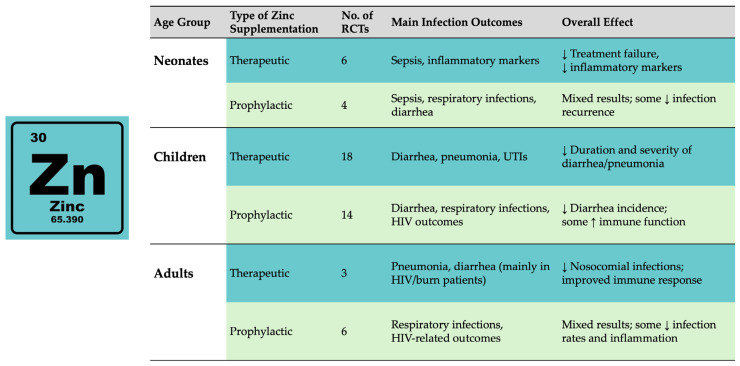
Summary of the current evidence about the relationship between zinc supplementation and infection, showing the age group, type of zinc supplementation, the number of included RCTs, main infection outcomes, and overall effect. Arrows indicate the direction of change over time (↑ increase; ↓ decrease).

After the research process using the described terms and a manual search of additional references, 51 Randomized Controlled Trials (RCTs) were included in our review: 27 for therapeutic zinc supplementation and 24 for prophylactic zinc supplementation (Figure 2).

### 2.2. Zinc Supplementation Against Infections in Neonates and Children

The effect of zinc differs substantially between therapeutic and preventive RCTs, with therapeutic supplementation showing the clearest benefits in septic neonates and preventive strategies demonstrating more heterogeneous results depending on baseline zinc status and population risk.

Table 1 describes the main RCTs on therapeutic and prophylactic zinc supplementation in neonates to date [12,13,14,15,16,17,18,19,20,21].

Irfan et al. summarized this evidence in a meta-analysis: therapeutic zinc supplementation during sepsis may significantly reduce treatment failure and potentially reduce the mortality rate [22]. Conversely, further randomized controlled trials are needed to support prophylactic zinc supplementation for preventing sepsis, given the different doses used [18,19].

Table 2 describes the main RCTs on therapeutic and prophylactic zinc supplementation in children to date [23,24,25,26,27,28,29,30,31,32,33,34,35,36,37,38,39,40,41,42,43,44,45,46,47,48,49,50,51,52,53,54]. There were no studies about therapeutic zinc supplementation in sepsis. Most studies aimed to evaluate the effects of therapeutic zinc supplementation in children with diarrhea, with a significant reduction in the episode’s duration or an increased immune response [23,24,25,26,28,30,31,32,33,36,40].

The aims of the studies about prophylactic zinc supplementation were heterogeneous, from the prevention of diarrhea to the increase in CD4+ cells in HIV-infected children [41,49,51].

### 2.3. Zinc Supplementation Against Infections in Adults

Table 3 describes the main RCTs on therapeutic and prophylactic zinc supplementation in adults to date [55,56,57,58,59,60,61,62,63]. The aims of the studies concerning therapeutic zinc supplementation were heterogeneous, from the risk of nosocomial pneumonia in patients with large thermal burns to the immune response in HIV-infected adults [55,56,57]. Conversely, the number of CD4+ cells in patients with HIV was the aim of most studies concerning prophylactic supplementation [61,62,63].

## 3. Discussion

An estimated 17% of the world’s population is at risk of insufficient zinc intake [2]. In particular, over half of preschool-aged children worldwide have micronutrient deficiencies, including zinc [64]. Furthermore, Littlejohn et al. showed that multiple micronutrient deficiencies (including zinc) in early life are associated with alterations in the gut microbiome and intrinsic antibiotic resistance genes in mice [65], highlighting micronutrient deficiency as a relevant public health concern. In this scoping review, we provided an integrated overview of RCTs evaluating zinc supplementation for the prevention and treatment of bacterial infections across the life course, from preterm neonates to adults.

### 3.1. Effects of Zinc Deficiency on Immune Function

Zinc is a key regulator of immune function; therefore, its deficiency can favor the development of a pro-inflammatory phenotype [66].

The maturation and functionality of T and B lymphocytes are negatively impacted by zinc deficiency. A well-known effect of zinc deficiency on T cells is a distorted Th1/Th2 balance, favoring Th2-driven allergic responses [60]. Zinc homeostasis is crucial for T-cell maturation and differentiation, and zinc supplementation may restore this dysfunction [60,67].

Older adults have been shown to exhibit dysfunctional adaptive immunity, characterized by pathologically increased interleukin-6 release, diminished T-cell activation, and reduced responses to stimulation or immunization [60,68,69].

Innate immunity can also be impaired by zinc deficiency, with negative effects on neutrophil functions (phagocytosis, oxidative burst, degranulation, cytokine production) [70] and natural killer (NK) cell function (decreased recognition of major histocompatibility complex class I on target cells with reduced zinc levels, as well as inhibitory killer-cell Ig-like receptor—KIR—polymerization in the presence of zinc) [66,71].

### 3.2. Zinc Supplementation Against Infections in Neonates and Children

The strongest and most consistent evidence for the anti-infective effects of zinc supplementation emerges in early life, when both zinc deficiency and infectious morbidity are highly prevalent. Combining 28 randomized controlled trials (including 237,068 participants), a meta-analysis revealed that zinc supplementation in children under 5 years significantly reduced the risk of all-cause mortality by 44%. Considering only low birth weight (LBW) infants, zinc supplementation reduced all-cause mortality by 52% [72].

From the first days of life, zinc appears to play a crucial role in defense against bacterial infections. Indeed, zinc deficiency has been demonstrated in neonates (especially if born preterm) and has been related to a higher infectious risk [73].

**Figure 3:**
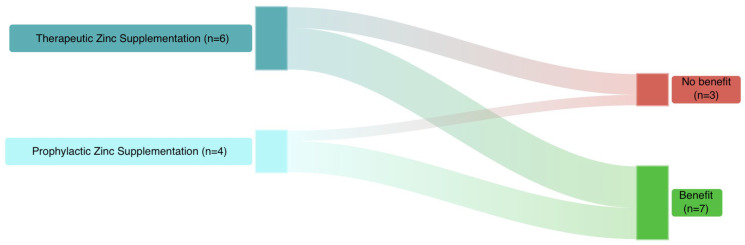
Summary of the current evidence from RCTs on zinc supplementation in neonates.

Available therapeutic RCTs in neonates showed that zinc supplementation supports the resolution of sepsis and improves outcomes. Overall, seven studies (70%) evaluating zinc supplementation reported beneficial effects, while three (30%) did not (Figure 3).

In older children, most studies evaluated the effects of therapeutic zinc supplementation in diarrhea, with a significant reduction in the episode’s duration or an increased immune response [23,24,25,26,28,30,31,32,33,36,40].

Moreover, a Cochrane review evidenced that the incidence of acute lower respiratory infections in children aged 2 months to 59 months was reduced by 35% (RR: 0.65, 95% CI: 0.52–0.82) with additional zinc intakes of less than 20 mg per day. More precisely, among the children who received zinc supplements, the incidence and prevalence of pneumonia were 13% (RR: 0.87, 95% CI: 0.81–0.94) and 41% (RR: 0.59, 95% CI: 0.35–0.99) lower, respectively [74].

**Figure 4:**
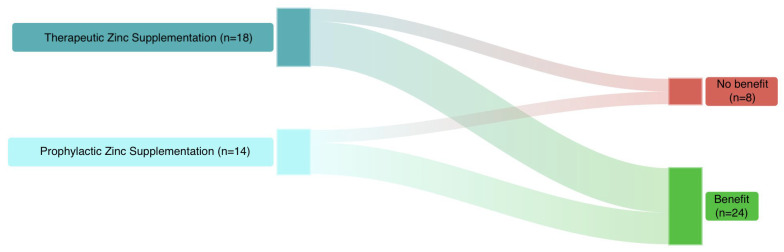
Summary of the current evidence from RCTs on zinc supplementation in children.

From available RCTs in children, twenty-four studies (75%) reported some benefits, while eight (25%) did not (Figure 4).

### 3.3. Zinc Supplementation Against Infections in Adults

Among adults, especially those with critical illness, HIV infection, or burn injuries, zinc supplementation has been associated with reduced nosocomial infections and improved immunological recovery, although findings remain heterogeneous across settings [55,56,57,58,60,62,63].

Furthermore, beyond available RCTs in the literature, which we previously summarized, several non-randomized studies also reported a potential role of zinc supplementation in preventing infections in adults. A better specific immune response has been identified in patients undergoing therapeutic zinc supplementation, in particular when zinc levels were low [55,56,57].

Lotfi et al. reported that end-stage renal disease patients on hemodialysis had lower serum zinc levels, which were associated with an increased risk of bacterial infection (although results were unadjusted for other covariates), compared to non-infected patients and healthy individuals. Catheter site infections and urinary tract infections were the most common infections [75].

Being one of the main causes of hospitalization and mortality for older people, particularly for those residing in nursing homes, pneumonia is a serious public health concern. In a prior clinical investigation, Meydani et al. showed that, compared to those with appropriate serum zinc levels, 29% of nursing home residents had low serum zinc levels (<70 mg/dL), which coincided with a two-fold increase in pneumonia incidence and duration, and increased antibiotic use, and overall mortality [76]. Ortega et al. are conducting a randomized, placebo-controlled, double-blind clinical pilot trial about zinc supplementation in the elderly to prevent pneumonia (NCT05527899). The results of this study will provide new insights concerning the safety and efficacy of zinc supplementation (30 and 60 mg/day elemental zinc), as well as the correlation between serum and cellular zinc concentrations [77].

Finally, Kiabi et al. found that zinc supplementation might be associated with a significant reduction in ventilator-associated pneumonia in adult mechanically ventilated trauma patients [78].

**Figure 5:**
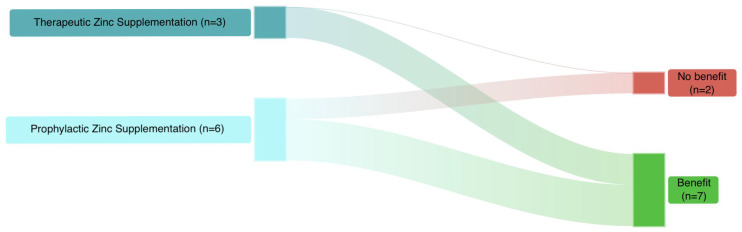
Summary of the current evidence from RCTs on zinc supplementation in adults.

Summarizing the few available RCTs in adults, seven studies (77.8%) reported some benefits, while two (22.2%) did not (Figure 5).

Across the included RCTs, zinc was administered in different formulations, including inorganic and organic salts. However, most trials did not directly compare different zinc forms, and dosing was primarily based on elemental zinc content. Therefore, for the purposes of this scoping review, zinc formulations were considered equivalent with respect to clinical support, as available evidence does not allow for definitive conclusions regarding differential bioavailability or efficacy on infection outcomes.

### 3.4. Zinc and Multi-Drug-Resistant Bacteria

Zinc ions have various physiological and biochemical functions, stabilizing the folded conformation of metalloproteins and participating in critical reactions. Zinc can interfere with bacteria in different ways, including zinc overloading and zinc deprivation. In the overloading approach, large volumes of zinc ions are released at the site of bacterial infection using zinc-loaded nanodelivery devices, interfering with bacterial transcriptional control, inactivating functional proteins, producing intracellular ROS and damaging bacterial DNA. Conversely, in the approach of zinc ion deprivation, zinc chelating agents cause an imbalance in zinc ion homeostasis by depriving intracellular zinc ions. Zinc-dependent enzymes become inactive, altering chromatin shape and preventing DNA replication, ultimately leading to bacterial death [79].

Zinc chelation can also potentiate antibiotic activity, as in the case of last-resort carbapenem antibiotics. They belong to the β-lactams class, and nearly all classes can be inactivated by zinc-requiring enzymes called metallo-β-lactamases (MBL) [80]. Bacteria encoding New Delhi MBL (NDM-1) enzymes are among the most fearsome because of the difficulty of treating these infections. Zinc-selective compounds could potentiate β-lactam antibiotics against an MBL-carrying pathogen by withholding zinc availability: Falconer et al. used a mouse model of NDM-1 infection, showing that combination treatment of the respective analog with meropenem resulted in a significant decrease in bacterial burden in contrast to animals that received antibiotic treatment alone [81].

Furthermore, a few bacterial zinc metalloenzymes are gaining interest as targets because they differ from endogenous human zinc-metalloproteinases in every way. The development of novel inhibitors against the enzymes involved in lipid A biosynthesis, bacterial nutrition, and sporulation -such as UDP-[3-O-(R)-3-hydroxymyristoyl]-N-acetylglucosamine deacetylase (LpxC), thermolysin (TLN), and pseudolysin (PLN)—has drawn increasing attention in recent decades from both industry and academia. However, it turns out that targeting these bacterial enzymes is more difficult than anticipated, and the dearth of viable clinical candidates implies that further work is required [82].

### 3.5. New Experimental Models with Zinc Nanoparticles Against MDR Bacteria

The biosynthesis of metallic nanoparticles has garnered increasing interest due to their favorable attributes, including low toxicity, cost-effectiveness, and ease of large-scale production. Among these, zinc oxide nanoparticles (ZnO-NPs) are particularly promising owing to their intrinsic photocatalytic and photo-oxidative properties, which confer potent antimicrobial activity. They should be considered an emerging and predominantly preclinical research direction rather than an extension of current clinical zinc supplementation strategies. Their relevance lies mainly in the context of antimicrobial resistance and novel anti-infective approaches, and they should be clearly distinguished from oral or systemic zinc supplementation evaluated in included randomized clinical trials.

The reduction in particle size and tailored surface functionalization further enhances their bactericidal efficacy against both Gram-positive and Gram-negative pathogens. Different microbes (bacteria, fungi, and yeast), and various parts of plants (leaf, root, fruit, flower, peel, etc.) have been exploited for the facile, rapid, cost-effective and non-toxic synthesis of ZnONPs [83].

Notably, ZnO-NPs have demonstrated significant potential in the prevention and control of infections caused by multidrug-resistant (MDR) bacteria [84]. The antibacterial efficiency of these particles can be high, yet at low doses (0.16–5.00 mmol/L) [85], with a high bactericidal potential [86].

There are different mechanisms of ZnO-NPs that can be listed: disruption of the cell membrane [87,88], binding to proteins and DNA, generation of reactive oxygen species (ROS) [89,90], disruption of the processes of bacterial DNA amplification, and down-regulation of expression in a wide range of genes [91].

Some authors propose them as an alternative to antibiotics against ESKAPE bacteria, hypothesizing a higher effectiveness and fewer side effects, although these findings should still be validated from a clinical point of view [92].

The literature on ZnO-NPs is rapidly expanding; however, nanoparticles synthesized using different physicochemical methods still face significant limitations that minimize their biocompatibility: the large-scale production of green synthesized ZnO-NPs remains the primary challenge for researchers [93].

#### 3.5.1. Gram-Positive Bacteria

Zinc oxide nanoparticles (ZNO-NPs) proved effective against Gram-positive bacteria such as Staphylococcus aureus, especially methicillin-resistant *Staphylococcus aureus* (MRSA) strains [94]. Punjabi et al. also assessed the synergistic action of nanoparticles in combination with a common antibiotic, gentamicin (590 g/mg), used to treat various bacterial infections by Checkerboard assay. Zinc oxide nanoparticles synergized with gentamicin only against MRSA but not against other Gram-positive and Gram-negative bacteria [94].

Nejabatdoust et al. evaluated the role of ZnO-NPs in modifying the antibacterial activity of ciprofloxacin (CIP): they found that ZnO-NPs showed a good bacterial inhibitory potential. Moreover, the simultaneous use of modified ZnO-NPs in combination with CIP significantly reduced the expression of the four efflux pumps norA, norB, norC, and tet38 by 5-, 3-, 2-, and 3-fold, respectively, compared to ciprofloxacin alone. Therefore, they suggested that zinc oxide nanoparticles, with their potent antimicrobial effects, could be used as an antimicrobial agent against *S. aureus* for preventive and/or therapeutic approaches [95].

Djearamane et al. examined the surface interaction between zinc nanoparticles and bacterial cell walls and the subsequent morphological alterations on the bacterial surface in two opportunistic pathogens, *Enterococcus faecalis* and *Serratia marcescens*. The growth-inhibitory test showed that ZnO-NPs had a dose-dependent growth-inhibitory impact on both test bacteria; the higher the nanoparticle concentration, the greater the suppression of bacterial growth, especially against *E. faecalis* [96].

#### 3.5.2. Gram-Negative Bacteria

Whereas some authors reported better results of ZnO-NPs against Gram-positive bacteria [97], others described a higher antibacterial effect against Gram-negative bacteria: probably the effect depends on the characteristics of nanoparticles and their biological synthesis [85]. For example, Elabbasy et al. recently synthesized zinc oxide nanoparticles with a potent, dose-dependent antibacterial and antibiofilm activity against selected MDR *E. coli* strains isolated from retail fish [98].

Similarly, Chauhan et al. demonstrated that ZnO-NPs could inhibit *Pseudomonas aeruginosa* [99].

Conversely, combining zinc nanoparticles and antibiotics can synergistically increase the antibacterial efficacy against Gram-negative bacteria, such as in the case of Colistin-resistant *Klebsiella pneumoniae*, where the synergistic activity of colistin combined with zinc oxide NPs and amikacin against colistin-resistant *K. pneumoniae* was found to be effective [100].

In the same way, Ghasemi and Jalal found that ZnO-NPs combined with ciprofloxacin and ceftazidime effectively treated *Acinetobacter baumannii*. A minimum inhibitory concentration (MIC) of 0.25 mg/mL ZnO-NPs boosted the antibacterial activity of both antibiotics by disrupting cell membranes and aggregating antibiotics inside the cells [101].

A key mechanism of zinc nanoparticles involves the disruption of bacterial cell membranes, with their penetration into the bacterial membrane leading to structural damage and eventual cell death [102]. Conversely, the different membrane composition of human cells lessens the possibility that zinc nanoparticles would damage them.

#### 3.5.3. Mycobacteria

Zinc nanoparticles could pierce the complex cell wall of mycobacteria, which is made up of various lipids and mycolic acids, resulting in membrane rupture and cell death.

According to first impressions by Heidary et al., silver and zinc oxide nanoparticles showed bacteriostatic effects against drug-resistant strains of *Mycobacterium tuberculosis* and thus may be considered promising anti-mycobacterial nano-drugs [103]. Zinc nanoparticles were found effective against *Mycobacterium tuberculosis* and its MDR strain, with an MIC of 1.25 mg/mL [94].

Geng et al. demonstrated that zinc oxide nanoparticles have biphasic roles on Mycobacterium-induced inflammation by increasing the levels of pro-inflammatory factors (activation of autophagy) and ferroptosis mechanisms in infected macrophages. Using ZnO-NPs improved the anti-Mycobacterium activity of ZnO-NPs in an in vivo mouse model and alleviated acute lung injury caused by ZnO-NPs [104]. These particles can have a synergistic interaction with antibiotics, such as in the case of rifampicin [105], and may act as potential antibacterial agents in future animal and clinical studies.

#### 3.5.4. Biofilm Formation

Bacteria or yeasts that are either Gram-positive or Gram-negative can form biofilms on indwelling catheters. It comprises microbial cells that are discharged into the extracellular space and encased in a self-secreting polymer matrix [106]. The matrix comprises water, proteins, lipids, polysaccharides, and extracellular DNA. In addition to acting as a barrier against the host’s immune defenses, this matrix can prevent antimicrobial medications from penetrating and offers protection from the external environment. Bacteria are gradually liberated from this biofilm, which prolongs the infection and encourages the spread of bacteria to other parts of the body. Because antimicrobial medications have difficulty penetrating the matrix, destroying the biofilm from the central venous catheter (CVC) is particularly difficult. Consequently, CVC removal is the gold standard approach in cases of catheter-related bloodstream infections that do not respond to systemic treatment [107,108,109].

ZnO-NPs can produce reactive oxygen species (ROS) when they encounter microbial cells, which can compromise the integrity of the biofilm matrix. According to Hajipour et al. (2012), these ROS, which include superoxide and hydroxyl radicals, induce oxidative damage to proteins, nucleic acids, extracellular polymeric substances (EPS), and other components of biofilms [110]. This destabilizes the biofilm and eventually results in microbial mortality.

Additionally, ZnO-NPs also have natural anti-adhesive qualities that prevent microorganisms from adhering to surfaces and, hence, stop the formation of biofilms. The electrostatic interactions between ZnO-NPs and microbial cell membranes are responsible for this anti-adhesive effect, which prevents early adhesion to host tissues or medical device surfaces [102].

In particular, Abdelghafar et al. noted that ZnO-NPs had an *S. aureus* antibiofilm effectiveness, with a synergistic antibiofilm effect of ZnO-NPs in combination with tested antibiotics (gentamycin, clindamycin, cefotaxime, ciprofloxacin, chloramphenicol, and azithromycin). ZnO-NPs were able to lower the hydrophobicity of the S. aureus cell surface, which may account for the observed decline in the capacity of the bacteria to form biofilms. When ZnO-NPs were applied to bacteria, the expression of genes linked to biofilm was much lower than in untreated cells [111].

### 3.6. Strengths and Limitations

This review summarizes randomized controlled trials (RCTs) on zinc supplementation across all age groups (from neonates to adults) focusing specifically on bacterial infections. A clear distinction was made between therapeutic and prophylactic zinc supplementation, offering a structured understanding of their respective clinical roles. The inclusion of 51 RCTs ensures a comprehensive overview, with robust evidence from diverse geographic and clinical settings.

Emerging therapeutic avenues, such as zinc nanoparticles and their role in combating antimicrobial resistance, were discussed to highlight future research directions.

Among limitations, the heterogeneity of study designs, zinc formulations, dosages, and outcome measures limited the possibility of performing a quantitative meta-analysis. Some included studies had small sample sizes or lacked detailed reporting on secondary outcomes such as laboratory or immune parameters. A key limitation of this scoping review is the absence of a formal risk-of-bias assessment of the included randomized trials, as the primary aim of this scoping review was to map and summarize available evidence rather than formally assess study quality. Furthermore, the evolving field of zinc nanoparticle research is still in preclinical or early clinical stages, with limited high-quality human data available to support firm conclusions.

## 4. Materials and Methods

### 4.1. Methodology

This scoping review was conducted according to the PRISMA-ScR (Preferred Reporting Items for Systematic reviews and Meta-Analyses extension for Scoping Reviews) guidelines. A completed PRISMA-ScR checklist is provided in Supplementary File S1. No review protocol was registered for this scoping review.

### 4.2. Eligibility Criteria

We included RCTs written in English and reporting clinical outcomes related to bacterial infections following zinc supplementation in neonates, children, or adults. We excluded observational studies, studies without infection-related outcomes, non-human studies, and trials where zinc was not administered as a distinct intervention. The time window (2000–1 November 2025) was chosen to capture contemporary dosing strategies and outcome definitions. Randomized controlled trials were specifically selected to minimize confounding and better assess causal relationships between zinc supplementation and infection-related outcomes. While this approach may limit the breadth of available evidence, we believe that it can increase internal validity and strengthen the interpretability of clinical effects.

### 4.3. Information Sources

We searched MEDLINE (PubMed) for eligible studies. The search covered the period from 2000, to 1 November 2025. Reference lists of included articles were also screened to identify additional relevant studies.

### 4.4. Search Strategy and Selection of Sources of Evidence

The PubMed search was conducted using the following terms: *“zinc” AND (“neonate” OR “infant” OR “child” OR “adult”) AND (“bacterial infection” OR “sepsis” OR “pneumonia” OR “diarrhea”).* Only English manuscripts and RCTs were considered. Two authors (D.U.D.R. and L.C.) independently assessed the study eligibility according to the pre-established criteria and performed an accurate check to exclude duplicates. Discussion with a third researcher (M.I.) resolved differences in opinion to achieve consensus.

### 4.5. Data Charting Process and Data Items

Data from each included RCT were charted using a standardized extraction form developed for this review. The form captured study design, population, intervention characteristics, dosing regimen, outcomes related to bacterial infections, and key findings. Data charting was conducted independently by two reviewers (D.U.D.R. and L.C.), and discrepancies were resolved through discussion with a third researcher (M.I.). For each study, we extracted the following variables: country and year, population characteristics (age group, clinical condition), type of supplementation (therapeutic or prophylactic), and main outcomes (e.g., sepsis, pneumonia, diarrhea, inflammatory markers, mortality).

### 4.6. Critical Appraisal of Individual Sources

Given the heterogeneity of study designs, populations, zinc formulations, and outcome measures, no quantitative synthesis or meta-analysis was performed, in line with the aims of a scoping review.

### 4.7. Outcomes

The objective was to map the breadth and nature of the available evidence on zinc supplementation and its role in bacterial infections across different age groups. Concerning the evidence from RCTs, our primary outcome was the effect of zinc supplementation on infections, whereas our secondary outcomes included laboratory changes. We focused specifically on bacterial infectious outcomes and distinguished between therapeutic and prophylactic zinc supplementation across age groups. Furthermore, we explored emerging research directions involving antimicrobial resistance and zinc-based nanotechnologies.

## 5. Conclusions

Zinc supplementation, both therapeutic and prophylactic, has demonstrated significant benefits in reducing the incidence, severity, and duration of infections, especially in neonates, children, and immunocompromised adults. With zinc deficiency still prevalent globally, especially in high-risk populations, its correction is essential for optimal immune function.

Beyond its immunomodulatory effects, zinc may play a key role in addressing antimicrobial resistance. Zinc oxide nanoparticles (ZnO-NPs) show promises as adjunct therapies due to their ability to disrupt bacterial growth, enhance antibiotic activity, and prevent biofilm formation.

Further research is needed to establish standardized dosing strategies to prevent and restore zinc deficiency and evaluate the short-term and long-term safety and efficacy of novel zinc-based interventions, including nanoparticles, in clinical settings.

## Figures and Tables

**Table 1:** Main RCTs on zinc supplementation reporting clinical outcomes about bacterial infections in neonatal age. Arrows indicate the direction of change over time (↑ increase; ↓ decrease).

First Author	Year, Country	Population	Intervention	Main Outcomes	Ref.
***Therapeutic Zinc Supplementation***					
Bhatnagar et al.	2012, India	Infants 7–120 days with probable SBI	Zinc 10 mg/day vs. placebo, in addition to standard antibiotics, until recovery	↓ Treatment failure (need to change antibiotics within 7 days, or need for ICU, or death)	[12]
Mehta et al.	2012, Nepal	Neonates with EOS/LOS	Zinc 1 mg/kg/day vs. placebo, in addition to standard antibiotics, until discharge/death	No differences in mortality or hospital stay	[13]
Banupriya et al.	2016, India	Neonates with clinical sepsis + ≥2 abnormal tests	Zinc 3 mg/kg twice daily vs. placebo, in addition to standard antibiotics for 10 days	No differences in hospital stay and mortality, but improved neurological status at one month of age	[14]
El Farargy et al.	2016, Egypt	Preterm neonates with probable sepsis	Zinc 3 mg/kg twice daily vs. placebo for 10 days, in addition to standard antibiotics	↓ Mortality (10% vs. 18%)	[15]
Elfarargy et al.	2022, Egypt	Preterm neonates with LOS	Zinc 1.4 mg/kg/day vs. placebo for 10 days, in addition to standard antibiotics	↓ CRP and PCT; ↓ Mortality (2/90 vs. 5/90); ↓ Treatment failure (12/90 vs. 20/90)	[16]
Wadhwa et al.	2025, India and Nepal	Infants 3–59 days with clinical severe infection	Zinc 10 mg/day vs. placebo for 14 days, in addition to standard antibiotics	No significant reduction in mortality and treatment failure	[17]
***Prophylactic Zinc Supplementation***					
Terrin et al.	2013, Italy	VLBW neonates or GA 24–32 weeks	Zinc 9 mg/day vs. placebo, up to discharge	No difference in LOS incidence, ↓ Morbidity (NEC) and mortality	[18]
Mathur and Agarwal	2015, India	Preterm neonates <7 days	Zinc 2 mg/kg/day vs. no zinc, up to 3 months CA	↓ Rehospitalizations; ↓ respiratory infections, diarrhea, sepsis	[19]
Habib et al.	2015, Pakistan	Healthy newborns ≤14 days	Zinc 10 mg vs. placebo for 18 weeks, along oral poliovirus vaccine administration	No bacterial sepsis reported in either group	[20]
Sahin et al.	2024, Turkey	VLBW preterm infants	Zinc 12 mg/day vs. standard multivitamin with zinc 3 mg/day, from enrollment to discharge	↓ NEC (1% vs. 11.2%) ↓ Culture-proven LOS ↓ Clinical sepsis	[21]

**Table 2:** Main RCTs on zinc supplementation reporting clinical outcomes about bacterial infections in children. Arrows indicate the direction of change over time (↑ increase; ↓ decrease).

First Author	Year, Country	Population	Intervention	Main Outcomes	Ref.
***Therapeutic Zinc Supplementation***					
Sazawal et al.	1995, India	Children 6–35 mo, acute diarrhea	Zinc 20 mg/day vs. placebo until recovery	↓ Risk of persistent diarrhea (−23%)	[23]
Bhutta et al.	1999, Pakistan	Children 6–36 mo, persistent diarrhea	Zinc 3 mg/kg/day versus placebo, for 28 days	No faster recovery	[24]
Penny et al.	1999, Peru	Children 6–36 mo, persistent diarrhea >14 days	Zinc 20 mg/day vs. placebo, for 14 days	↓ Duration of persistent diarrhea	[25]
Baqui et al.	2002, Bangladesh	Children 3–59 mo, diarrhea	Zinc 20 mg/day vs. none, for 14 days	↓ Duration; ↓ mortality; ↓ hospital admissions	[26]
Brooks et al.	2004, Bangladesh	Children 2–23 mo, severe pneumonia	Zinc 20 mg/day vs. placebo, until discharge	Faster recovery; ↓ complications; ↓ antibiotic exposure	[27]
Raqib et al.	2004, Bangladesh	Children 12–59 mo, who had bloody mucoid stools and *Shigella* spp.	Zinc 20 mg/day + multivitamins vs. multivitamins, for 14 days	↑ Lymphocyte proliferation; ↑ Shigella-specific IgG	[28]
Mahalanabis et al.	2004, India	Children 2–24 mo, severe ALRI	Zinc 20 mg/day ± vit. A vs. placebo, for 5 days	↓ Fever duration and severity (boys only)	[29]
Patel et al.	2005, India	Children 6–59 mo, acute diarrhea	Oral rehydration solution (ORS) + zinc/copper vs. standard ORS, until recovery	↓ Diarrhea complications	[30]
Roy et al.	2007, Bangladesh	Children 3–24 mo, diarrhea	Zinc 20 mg/day in a multivitamin syrup vs. placebo, for 14 days	↓ Stool output and duration.	[31]
Patel et al.	2009, India	Children 6–59 mo, acute diarrhea	Zinc alone 20 mg/day vs. zinc + copper vs. placebo, for 14 days	No benefit observed	[32]
Dutta et al.	2011, India	Children 6–24 mo, diarrhea	Zinc 20 mg/day ± micronutrients vs. placebo, for 14 days	Zinc alone as effective as combinations	[33]
Shah et al.	2012, Nepal	Children 2 mo–5 yrs, severe pneumonia	Zinc 10 mg/day vs. placebo, for 7 days	No ↓ in pneumonia duration or hospital stay	[34]
Shah et al.	2013, India	Children 6–59 mo, ALRI	Zinc 10 mg/day vs. placebo	↓ Respiratory morbidity (shorter recovery)	[35]
Yazar et al.	2016, Turkey	Children 6–120 mo, acute diarrhea	Zinc 15 mg/day for 5 days vs. synbiotics (probiotics, prebiotics, vitamins) for 5 days vs. standard therapy	↓ Duration of diarrhea (both zinc + synbiotics)	[36]
Yousefichaijan et al.	2016, India	Children with UTIs	Zinc + standard therapy vs. standard therapy (not available dose and duration)	Faster recovery, but abdominal pain was exacerbated by zinc and lasted longer	[37]
Howie et al.	2018, Gambia	Children 2–59 mo, severe pneumonia	Zinc (infants: 10 mg/day; children: 20 mg/day) vs. placebo, for 7 days	No benefit in treatment failure	[38]
Rerksuppaphol and Rerksuppaphol	2019, Thailand	Hospitalized children with pneumonia	Zinc 15 mg/day vs. placebo, for 7 days	Faster resolution; shorter hospital stay	[39]
Dhingra et al.	2020, India and Tanzania	Children 6–59 mo, diarrhea	Zinc 5–20 mg/day vs. placebo, for 10–14 days	Low-dose = non-inferior; ↓ vomiting	[40]
***Prophylactic Zinc Supplementation***					
Rosado et al.	1997, Mexico	Children 18–36 mo	Zinc 20 mg/day vs. iron vs. both vs. placebo, for 12 months	↓ Episodes of infectious disease	[41]
Ruel et al.	1997, Guatemala	Children 6–9 mo	Zinc 10 mg/day vs. placebo, for 7 months	↓ Diarrhea incidence (−22%)	[42]
Sazawal et al.	1998, India	Children 6–35 mo	Zinc 10 mg/day vs. placebo, for 6 months	↓ Acute lower respiratory infections (–45%)	[43]
Bobat et al.	2005, South Africa	HIV-infected children	Zinc 10 mg/day vs. placebo, for 6 months	↓ Diarrhea morbidity; no ↑ viral load	[44]
Long et al.	2006, Mexico	Children 6–15 mo	Zinc 20 mg/day vs. vitamin A vs. combined vs. placebo, for 12 months	Zinc ↓ diarrhea in select subgroups	[45]
Abdulhamid et al.	2008, USA	Children 7–18 mo. with cystic fibrosis	Zinc 30 mg/day vs. placebo, for 12 months	↓ Respiratory infection antibiotic days	[46]
Consolo et al.	2013, Brazil	Children 1–18 yrs	Zinc 2 mg/kg/day vs. placebo, for 60 days	↓ Infections despite no ↑ in plasma zinc	[47]
Malik et al.	2013, India	Infants 6–11 mo	Zinc 20 mg/day vs. placebo, for 14 days	↓ Episodes (−39%) and duration of diarrhea (−36%)	[48]
Lodha et al.	2014, India	HIV-infected children > 6 mo	Zinc 20 mg/day vs. placebo, for 24 weeks	No effect on CD4, viral load, or morbidity	[49]
McDonald et al.	2015, Tanzania	Infants > 6 w. born to HIV-negative mothers	Multivitamins + zinc 5 mg vs. placebo, for 18 months	↓ Diarrhea and URTI; ↑ mortality trend (not-significant)	[50]
Martinez-Estevez et al.	2016, Colombia	Children 6–12 mo	Zinc 5 mg/day vs. placebo, for 12 months	↓ URTI and diarrheal episodes	[51]
Sharma et al.	2016, India	Children 5–15 yrs, cystic fibrosis	Zinc 30 mg/day vs. placebo, for 12 months	No differences in infections or function	[52]
Carcillo et al.	2017, USA	Children 1–18 yrs, critical illness risk (3 groups: healthy, immune-competent with lymphopenia and immunocompromised infants)	Zinc 20 mg/day + selenium + glutamine + metoproclamide vs. protein powder, during PICU stay	No effect on nosocomial infection/sepsis	[53]
Namazzi et al.	2023, Uganda	Children with sickle cell anemia	Zinc 10 mg/day vs. placebo, for 12 months	No prevention of severe infections	[54]

**Table 3:** Main RCTs on zinc supplementation reporting clinical outcomes about bacterial infections in adults. Arrows indicate the direction of change over time (↑ increase; ↓ decrease).

First Author	Year, Country	Population	Intervention	Main Outcomes	Ref.
***Therapeutic Zinc Supplementation***					
Berger et al.	2006, Switzerland	Adults 16–65 yrs with major burns	Intravenous trace element supplements (copper, selenium, and zinc 26–31 mg/day) vs. placebo, for 8–21 days	↓ Nosocomial pneumonia; ↑ antioxidant status	[55]
Baum et al.	2010, USA	HIV-infected adults with low plasma zinc levels	Zinc (women: 12 mg; men: 15 mg) vs. placebo, for 18 months	↓ Immunologic failure; ↓ diarrhea episodes	[56]
Ejemot-Nwadiaro et al.	2019, Nigeria	Adults with Tuberculosis (TB)	Zinc 25 mg/day + TB therapy vs. TB therapy alone, for 60 days	Trend toward ↓ acid-fast bacilli positivity (RR ~0.59)	[57]
***Prophylactic Zinc Supplementation***					
Ertekin et al.	2003, Turkey	Adults receiving radiotherapy	Zinc 50 mg/day vs. placebo, for 6 weeks	↓ Oropharyngeal infections (*Candida*, *Staphylococci*)	[58]
Deloria-Knoll et al.	2006, USA	HIV-positive injection drug users (≥18 years)	Zinc 50 mg/day vs. Vitamin A vs. Zinc + Vitamin A vs. placebo before immunization with heptavalent pneumococcal glycoprotein conjugate vaccine (PC-7)	No enhancement of vaccine immunogenicity	[59]
Prasad et al.	2007, USA	Adults 55–87 yrs	Zinc 45 mg/day vs. placebo, for 12 months	↓ Infections; ↓ TNF-α and oxidative stress	[60]
Asdamongkol et al.	2013, Thailand	HIV-infected adults with immunologic discordance	Zinc 15 mg/day vs. placebo, for 6 months	↑ CD4+ count in low-zinc individuals	[61]
Hadadi et al.	2020, Iran	Patients with HIV	Zinc 50 mg/day vs. selenium vs. placebo, for 6 months	↓ Opportunistic infections with zinc	[62]
Silva et al.	2021, Chile	Patients with HIV with immunovirological discordance	Zinc 15 mg/day vs. placebo, for 12 months	No significant differences in CD4+ levels	[63]

## Data Availability

No new data were created or analyzed in this study. Data sharing is not applicable to this article.

## Supplementary Materials

The following supporting information can be downloaded at: https://www.mdpi.com/article/10.3390/antibiotics15010066/s1, File S1: PRISMA-ScR-Fillable-Checklist. Ref. [112] is cited in the Supplementary Materials.

## Abbreviations

The following abbreviations are used in this manuscript:

ALRI	Acute lower respiratory infection
CA	Corrected age
CF	Cystic fibrosis
CIP	Ciprofloxacin
CI	Confidence interval
CRP	C-reactive protein
CVC	Central venous catheter
EOS	Early-onset sepsis
EPS	Extracellular polymeric substances
GA	Gestational age
HIV	Human immunodeficiency virus
IL-6	Interleukin-6
KIR	Killer-cell Ig-like receptor
LBW	Low birth weight
LOS	Late-onset sepsis
LpxC	UDP-[3-O-(R)-3-hydroxymyristoyl]-N-acetylglucosamine deacetylase
MBL	Metallo-β-lactamase
MDR	Multidrug-resistant
MHC-I	Major histocompatibility complex class I
MIC	Minimum inhibitory concentration
MRSA	Methicillin-resistant *Staphylococcus aureus*
NCT	ClinicalTrials.gov identifier
NDM-1	New Delhi metallo-β-lactamase 1
NEC	Necrotizing enterocolitis
NK cells	Natural killer cells
PICU	Pediatric intensive care unit
PCT	Procalcitonin
PLN	Pseudolysin
PRISMA-ScR	Preferred Reporting Items for Systematic reviews and Meta-Analyses extension for Scoping Reviews
RCT/RCTs	Randomized controlled trial/Randomized controlled trials
ROS	Reactive oxygen species
RR	Relative risk
SBI	Serious bacterial illness
SCA	Sickle cell anemia
SEM	Scanning electron microscopy
TB	Tuberculosis
Th1/Th2	T helper 1/T helper 2
TLN	Thermolysin
URTI	Upper respiratory tract infection
Zn	Zinc
Zn^2+^	Zinc ion
ZnO-NPs	Zinc oxide nanoparticles
ZnuA/ZnuB/ZnuC	Components of the ZnuABC zinc import system
ZIP14	Zinc transporter protein 14
